# Present and Future of the Poor-Law Infirmary

**Published:** 1913-05-10

**Authors:** 


### PRESENT AND FUTURE OF THE POOR-LAW INFIRMARY.

### Poor Law and Research.

By H. B. M.

The recent decision of the Metropolitan Asylums Board to appoint a research bacteriologist was welcome news to the advocates of a more progressive spirit in medicine. But the Local Government Board has suggested that the officer to be appointed should be known as "pathologist," and that, at least for the present, he should not give his whole time to the duties of the office. A retrograde step of this description would nullify the character of the scheme. A pathologist, clinical or otherwise, is not an urgent necessity. A keen research bacteriologist whose whole time can be devoted to investigating the epidemiology of infectious disease is needed. Such an appointment may lead to an extensive scheme of research later. This research question was pressed in Dr. Birdwood's article on administration in The Hospital of January 6, 1912.

At present the Metropolitan Asylums Board simply house, isolate, and treat persons suffering from the infectious epidemic diseases and some of the Poor-Law children. Research work and investigation as to the causes of these diseases have seldom been encouraged. Material lies plentiful to hand, and it is left untouched. To be fair, however, one must mention this fact: In all rate-supported institutions—and it is an important point of distinction between these institutions and the voluntary hospitals—investigation is not encouraged because of the popular prejudice against research, or, as it is termed, "experiment." The investigator as a resident medical officer is not wanted because of fear of his methods, and of the difficulty of knowing when his researches are transgressing the bounds of permissibility. The public, as represented by the citizen unit, is prepared to allow a little manipulation, say, to allow a blood count to be made, while resident in an institution depending on the generosity of the wealthy; but when at home in an institution which its rates help to support it stipulates "treatment with as little discomfort as possible."

Hence, in spite of the abundance of excellent clinical material and the extensive field open to those in quest of knowledge, the comparatively infinitesmal amount of work and published records which has issued from Poor-Law infirmaries cases interesting, rare, and sometimes unique, are left unrecorded; the pay of the resident medical officer is small; the institution is always understaffed; the best men are seldom secured; and when a keen resident does attempt to do even the ordinary routine work of his training school or the commonplaces of the voluntary hospitals, he finds himself handicapped—in the first place, by the poor equipment of the institution, and, secondly, by the great homage to a supposed popular prejudice against research or experiment. Even in some of the Metropolitan Poor-Law infirmaries a microscope is not provided, not to mention a hæmocytometer or a sphygmomanometer. Patients are operated on for appendicitis, treated for pneumonia, given vaccines and anti-sera without a blood count having been once taken, and that in a supposed modern building.

The Metropolitan Asylums Board, however, are an exceptional body. They represent the cream of Poor-Law authorities and more, consisting as they do of selected representatives from the various elected Metropolitan Boards of Guardians, plus a nominated element. They have an almost world-wide reputation for administrative efficiency in infectious diseases. Their institutions are always well equipped with a modern laboratory and all the modern scientific implements; yet because of this discouragement of investigation little or nothing emanates from their institutions. True an annual report, mainly statistical, is issued, but recorded clinical observations and investigations are rarities. Even if work of a confirmatory nature was done and issued, it would be breaking the ice of research apathy. The experimental or research work or investigation may entail absolutely no discomfort to a patient.

It is a moot question whether the idea of an existence of a popular prejudice against experiment has any basis in fact. If it does exist, the sooner it is removed the better. A well-directed sustained effort in the study of the epidemiology and causation of the infectious fevers would result in a great saving.

It is folly to spend money in the proper equipment of an institution if no attempt must be made to utilise the admirable instruments available. Some stimulus ought, therefore, to be given to this question of research or "work done" in all Poor-Law institutions, and especially the modern buildings under the control of the Metropolitan Asylums Board, where facilities exist for such work, and where the resources of the Metropolitan Asylums Board laboratories at Sutton may be drawn on to some extent. The following suggestions may be made:—

1. That prizes, of medals or books, be annually offered by the Metropolitan Asylums Board for investigation work done by the resident medical officers of their institutions.

2. That medical superintendents stipulate on the appointment of such officers that, in addition to their ordinary routine clinical work, some investigation, the nature of which may be decided on by the investigator, be pursued—a record of the investigation to be forwarded within a limited period.

3. That resident medical officers be encouraged to undertake investigation and even to apply for grants and scholarships such as those granted by the British Medical Association.

4. That the Metropolitan Asylums Board insist, in answer to the Local Government Board, that their forthcoming appointment should be a full-time research bacteriologist for studies in epidemiology, etc., and not a part-time "pathologist."

